# A Case of Postoperative Sepsis Triggered by Fecal Retention at the Suture Site After Appendectomy

**DOI:** 10.1155/cris/6025864

**Published:** 2025-12-01

**Authors:** Hirotaka Kato, Makoto Seki, Atomu Katayama, Masakazu Yoshida

**Affiliations:** Department of Surgery, Mitaka Central Hospital, 5-23-10 Kami-Renjaku, Mitaka, Tokyo 181-0012, Japan

**Keywords:** acute appendicitis, appendectomy, postoperative sepsis

## Abstract

Postoperative sepsis after appendectomy can sometimes be led by surgical site infection, intra-abdominal abscess, or intestinal obstruction. However, there have been no reports that postoperative sepsis is certainly caused by only fecal retention in the intestine, including the appendectomy stump. A 60-year-old healthy woman visited a doctor with a chief complaint of right lower abdominal pain. Abdominal computed tomography (CT) showed a swollen appendix with fecal calculus, and then the patient was diagnosed with acute appendicitis. The patient underwent a laparoscopic appendectomy the next day. The appendix was resected at the level of the appendicular root with an endostapler. The patient was discharged from the hospital on postoperative day (POD) 4 in a good general condition. However, the patient visited the hospital on POD 15 with a chief complaint of fever. A medical interview revealed a decrease in the number of bowel movements compared to before the appendectomy. The quick Sequential Organ Failure Assessment (SOFA) score with the vital signs showed two points, and the SOFA score with the blood examination showed a total increase of four points compared to the previous blood examination. The patient was therefore suspected of sepsis after appendectomy. Abdominal CT showed obvious fecal retention in the ileocecal region, including the appendectomy site. Furthermore, abdominal contrast CT did not reveal any obvious thrombosis in the portal venous system. With conservative treatment by antibiotics and laxatives, the fever gradually resolved, and the patient was discharged on POD 27. Abdominal radiography showed no findings of fecal retention. The patient has had regular bowel movements and has not experienced a recurrence of the symptoms. Postoperative sepsis might be caused even in healthy patients. It was conceivable that postoperative assessment of bowel movements was necessary to detect the risk of postoperative sepsis.

## 1. Introduction

Postoperative sepsis after appendectomy can be led particularly by surgical site infection, intra-abdominal abscess, or intestinal obstruction [1]. Other conditions to consider that may cause postoperative sepsis after appendectomy and require differential diagnosis include hematogenous infection associated with severe preoperative appendicitis [2], bacterial translocation due to postoperative intestinal ischemia [3], and local inflammation caused by fecaliths or fecal retention remaining at the appendiceal stump [4]. However, there have been no any previous reports that postoperative sepsis occurring in healthy individual after appendectomy is certainly caused by only fecal retention in the intestine, including the appendectomy stump.

Herein, we report a case in which a 60-year-old healthy woman who underwent laparoscopic appendectomy for acute appendicitis developed a fever 15 days after surgery, and abdominal computed tomography (CT) showed only fecal retention in the ileocecal region, including the appendectomy stump, except for which any causes that led to postoperative sepsis could not be found.

## 2. Case Presentation

**Figure 1:**
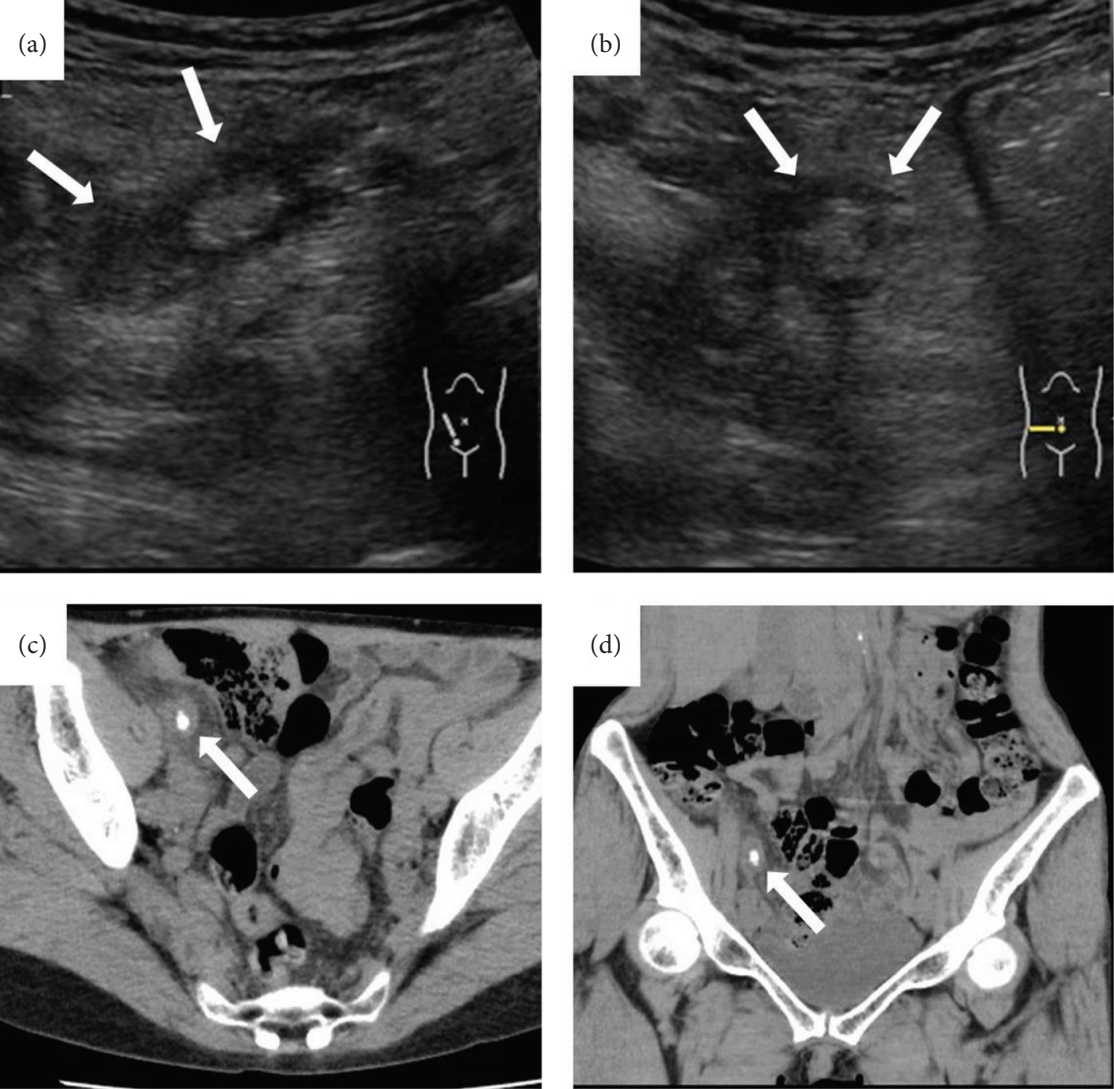
Abdominal ultrasonography and abdominal CT findings on preoperation. (a, b) Abdominal ultrasonography showed a swollen appendix and fecalith in the appendiceal lumen (arrows). (c, d) Abdominal CT also showed swelling of the appendix with fecalith and a small amount of fluid collection around the appendix (arrows) but no obvious free air.

A 60-year-old healthy woman visited a local doctor with a chief complaint of right lower abdominal pain. An abdominal CT showed a swollen appendix with fecal calculus, and then the patient was diagnosed with acute appendicitis and referred to our hospital for treatment. The patient had no medical history and was not taking any medications. Vital signs showed a respiratory rate of 18 breaths, SpO2:97% (room air), pulse: 63 beats/min, blood pressure: 112/68 mmHg, and body temperature: 37.8°C. Physical examination revealed a soft abdomen except for localized tenderness and recurrent pain at the right lower abdomen. Blood examination described as preoperation showed elevated white blood cell (WBC) and C-reactive protein (CRP) as shown in Table 1. Abdominal ultrasonography showed a swollen appendix with fecalith (Figure 1a,b). Abdominal CT showed swelling of the appendix with fecalith and a small amount of fluid collection around the appendix but no obvious free air (Figure 1c,d). The patient was diagnosed with acute appendicitis, and a laparoscopic appendectomy was planned for the next day.

### 2.1. Operative Finding

**Figure 2:**
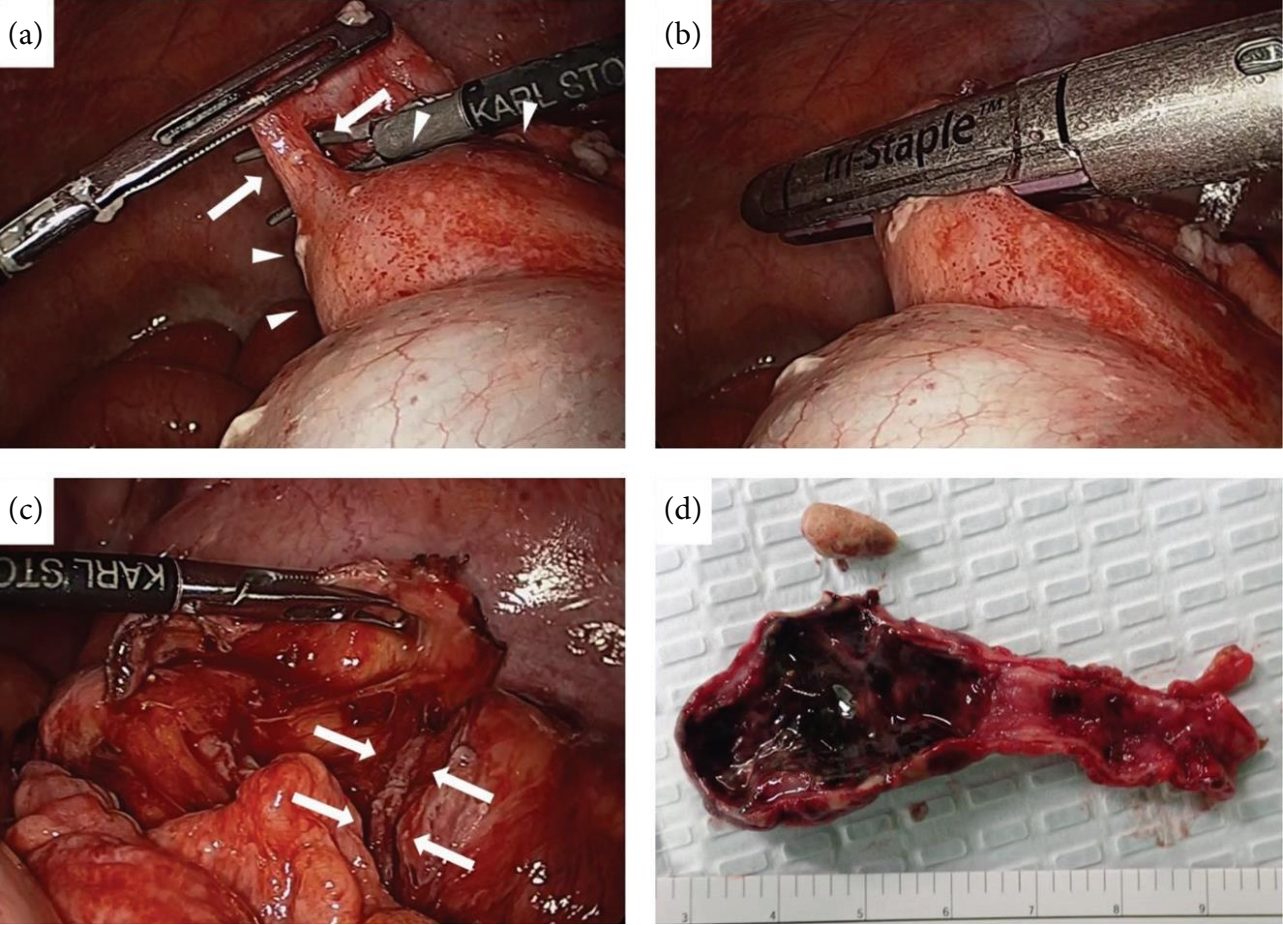
Operative and specimen findings. (a) Orientation of the appendicular root (arrows) and ileocecal region (arrowheads). (b) The appendix was resected at the level of the appendicular root with an endostapler. (c) The appendicular transection stump (arrows) was covered with peripheral tissue of the appendix. (d) There was an ulcer lesion in the mucosa but no obvious perforation.

Laparoscopic surgery was performed with three ports placed in the umbilicus, right side of the abdomen, and left lower abdomen, respectively. There was no obvious contaminated ascites in the abdominal cavity. A swollen appendix was identified, and a fluid collection was observed around the swollen appendix. The appendix was adherent to the cecum due to inflammation. The appendix was separated at the level of the appendicular root with an endostapler (Figure 2a,b), and the appendicular transection stump was covered with peripheral tissue of the appendix (Figure 2c). The abdominal cavity was lavaged, and the wound was closed without drains.

### 2.2. Resection Specimen and Pathological Findings

A fecalith was found in the appendix lumen, and an ulcer lesion was observed in the mucosa. There was no obvious perforation (Figure 2d). Although severe invasion of neutrophils was observed throughout the entire appendix wall, no obvious malignant findings were observed.

### 2.3. Postoperative Course

**Figure 3:**
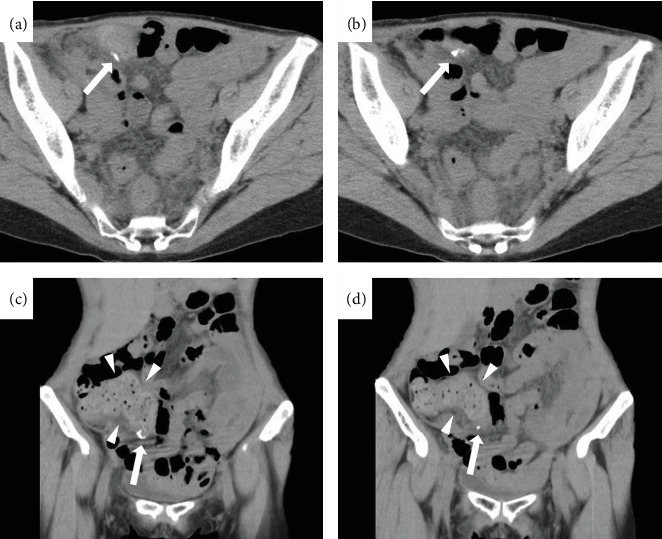
Abdominal CT findings on POD 15. (a–d) Abdominal CT showed no fluid collection around the appendectomy site (arrows), but significant fecal retention in the ileocecal region, including the appendectomy site (arrowheads).

**Figure 4:**
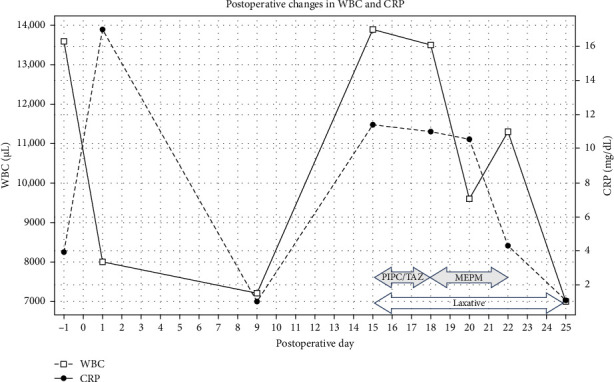
Postoperative changes in WBC and CRP. The blood examination on the first visit with a chief complaint of right lower abdominal pain described as POD −1 showed elevation of WBC: 13,600/μL and CRP: 3.91 mg/dL. The blood examination at outpatient follow-up on POD 9 showed a reduction of WBC: 7200/μL and CRP: 1.01 mg/dL. The blood examination on POD 15 with a chief complaint of fever showed re-elevation of WBC: 13,900/μL and CRP: 11.31 mg/dL. PIPC/TAZ was indicated for postoperative sepsis from POD 15 to POD 18. PIPC/TAZ was switched to MEPM at POD 18 because the Eubacterium spp. was resistant to PIPC/TAZ. After then, the blood examination showed a gradual decrease in WBC and CRP with antibiotics and laxatives.

The patient started drinking water and eating on postoperative day (POD) 1, and the patient was in a good general condition with a tendency toward fever reduction and was discharged on POD 4. The outpatient follow-up of POD 9 presented no episodes of fever or abdominal pain after discharge from the hospital and no significant inflammatory findings on blood examination, as shown in Table 1. However, the patient visited the hospital on POD 15 with a chief complaint of fever. A medical interview revealed a decrease in the number of bowel movements compared to before the appendectomy. Vital signs showed respiratory rate: 24 breaths/min, SpO2: 98%, pulse: 93 beats/min, blood pressure: 89/57 mmHg, and body temperature: 38.6°C. The vital sign revealed a quick sequential organ failure assessment (SOFA) score of two points, including one point for systolic blood pressure less than 100 mmHg and one point for respiratory rate more than 22 breaths/min. The physical examination revealed no redness or swelling at the wound site and no obvious physical findings, including abdominal tenderness or rebound tenderness. The blood examination showed elevated WBC and CRP levels, as shown in Table 1. The SOFA score showed a total increase of four points, including two points for platelet (PLT), one point for total bilirubin (T-BIL), and one point for creatinine (Cre). Urinalysis showed negative for protein, sugar, and occult blood, and urine sediment showed 1–4 red blood cells/high power field (HPF), 1–4 WBC/HPF, and negative for bacteria. The abdominal ultrasonography showed no fluid collection around the appendectomy site or any other intra-abdominal infectious lesions or thrombosis. Abdominal CT showed no fluid collection around the appendectomy site but significant fecal retention in the ileocecal region, including the appendectomy site (Figure 3). Furthermore, abdominal contrast CT did not reveal any obvious thrombosis in the intraperitoneal portal venous system. The patient was suspected of having sepsis after appendectomy because a quick SOFA score on POD 15 had two points, or the SOFA score on POD 15 had increased by two points or more compared to POD 9. After blood cultures were obtained, conservative treatment with antibiotics and laxative control was initiated. The Eubacterium spp., detected by anaerobic blood culture, showed resistance to piperacillin/tazobactam (PIPC/TAZ). The antibiotic was switched to meropenem (MEPM). After then, the fever gradually resolved, and follow-up blood cultures presented negative. The blood examination showed a decrease in inflammatory findings (Figure 4), and the patient was discharged on POD 27 with a good general condition. Abdominal radiography showed no findings of fecal retention around appendectomy site. The patient has had regular bowel movements and has not experienced a recurrence of symptoms since being discharged from the hospital.

## 3. Discussion

Postoperative sepsis, as one of the complications after appendectomy, was mainly caused by surgical site infection, intra-abdominal abscess, or intestinal obstruction [5] and typically presents a fever [6]. Most cases of postoperative sepsis occur within 30 days after appendectomy, with a reported mortality rate of 5.5% [1]. Pathologies leading to postoperative sepsis include direct dissemination sepsis, where bacteria spread from a localized site to the systemic circulation due to intra-abdominal abscesses or stump perforation, and intestinal sepsis, which arises from intestinal barrier failure mediated by mucosal ischemia and edema caused by intestinal content stasis and increased lumen pressure due to fecal retention or fecaliths [7]. The causes of postoperative sepsis raised from disease-related or procedure-related risk factors included preoperative management and surgical techniques, including bowel preparation, the use of antibiotics, fecal contamination in the case of emergent surgery, and tension applied at the sutures or anastomoses [8, 9]. On the other hand, postoperative sepsis raised from patient-related risk factors associated highly with age over 65 years and comorbidities including malignancies, chronic obstructive pulmonary disease, liver disease, diabetes, cardiovascular disease, morbid obesity, and renal failure or dialysis [1, 10]. The current patient developed a fever on POD 15 and had two points in the quick SOFA score with vital signs. Although the present patient was a 60-year-old woman without underlying diseases and had no patient-related risk factors for postoperative sepsis, possible causes of fever 2 weeks after surgery included intra-abdominal abscess, peritonitis due to intestinal leakage, and surgical site infection. Physical examination revealed no redness or swelling at the wound site, and there was no obvious tenderness or rebound tenderness. Blood examination showed significantly elevated levels of inflammatory reaction and an increase of more than two points in the SOFA score compared to the blood examination on POD 9, leading to a suspicion of sepsis [11]. An abdominal CT showed no obvious fluid collection around the appendectomy site or below the wound. Therefore, intra-abdominal abscess, peritonitis due to intestinal leakage, and surgical site infection, which could be the causes of postoperative fever, were ruled out. Urinary tract infections and respiratory infections were listed as possible causes of postoperative fever other than complications from appendectomy, but urine examination and chest CT showed no findings of urinary tract infection or respiratory infection.

As for procedure-related risk factors for postoperative sepsis, the modality of endostaplers for resecting the appendix can also be associated with postoperative complications, including intra-abdominal abscess or sepsis [12–14]. In the current patient, although the base of the appendix was resected with an endostapler, no exposure of intestinal contents or intestinal damage was observed. One of the conditions that causes fever approximately 2 weeks after appendectomy can include thrombosis. There was a report of thrombophlebitis that developed from superior mesenteric vein thrombosis about 2 weeks after appendectomy [15], being associated with fatal complications such as sepsis or septic embolism [16]. Although the intraperitoneal venous system was evaluated by abdominal ultrasound and abdominal contrast CT for postoperative fever or sepsis, no significant thrombosis was detected. The current blood cultures on POD 15 detected the genus Eubacterium, a type of enterobacteria, similar to previous reports on appendectomy [17], which has been linked to postoperative sepsis and disseminated intravascular coagulation (DIC) [18, 19]. Considering the abdominal CT findings of fecal retention in the intestinal tract, including the appendectomy site, and the detection of enterobacteria in the blood culture, the cause of postoperative fever and sepsis was thought to be bacterial translocation due to elevation in intestinal pressure due to fecal retention in the intestine, including the suture site. When the mucosal barrier at the intestinal tract including the appendix is compromised, bacterial and endotoxin may migrate to mesenteric lymph nodes, the portal venous system, and systemic circulation [20, 21]. In the current patient, pathological severe inflammation findings throughout the entire appendix were observed. The severe inflammation may have caused temporary weakening of postoperative intestinal movement, leading to stool retention and constipation, which caused blood flow disorders at the intestinal wall, allowing the bacteria to enter the bloodstream. The conservative treatment with antibiotics sensitive to Eubacterium spp. and laxative administration alongside follow-up with abdominal radiography examination to monitor intestinal function was considered useful in treating the condition.

## 4. Conclusions

Postoperative sepsis might be caused by elevation of the intestinal pressure due to fecal retention in the intestines, including the appendectomy site, even in healthy patients without risk factors. It was conceivable that postoperative assessment of bowel movements through patient interviews and evaluation of fecal retention via abdominal radiography examination was necessary to detect the risk of postoperative sepsis.

## Figures and Tables

**Table 1:** Blood examination on preoperation, POD 9 and POD 15.

Items	Preoperation	POD 9	POD 15
WBC (/μL)	13,600	7200	13,900
RBC (10^4^/μL)	449	363	383
Hb (g/dL)	13.9	11.1	11.5
Ht (%)	40.7	33.3	34.3
PLT (10^4^/μL)	18.0	32.1	8.9
PT (%)	100	100	88
PT-INR	0.90	0.95	1.24
APTT (s)	30.8	28.6	43.6
TP (g/dL)	7.3	5.9	6.7
Alb (g/dL)	4.5	2.9	3.1
CK (IU/L)	72	48	35
AST (IU/L)	24	17	113
ALT (IU/L)	20	23	111
LDH (IU/L)	200	143	144
ALP (IU/L)	77	59	72
γGTP (IU/L)	21	28	23
T-Bil (mg/dL)	1.1	0.5	1.5
AMY (IU/L)	125	68	66
Cre (mg/dL)	0.72	0.67	1.24
BUN (mg/dL)	19.4	8.4	44.1
eGFR (mL/min)	63.1	68.3	36.3
Na (mEq/L)	141	141	137
K (mEq/L)	3.8	4.2	3.5
Cl (mEq/L)	106	106	102
CRP (mg/dL)	3.91	1.01	11.31

*Note:* Blood examination on preoperation showed elevated WBC; 13,600/μL and CRP; 3.91 mg/dL. No significant inflammatory findings on POD 9. The blood examination on POD 15 showed elevated WBC; 13,900/μL and CRP 11.31 mg/dL. The SOFA score at POD 15 showed a total increase of four points, including two points for PLT, one point for T-BIL, and one point for Cre compared to POD 9.

Abbreviations: Alb, albumin; ALP, alkaline phosphatase; ALT, alanine aminotransferase; AMY, amylase; APTT, activated partial thromboplastin time; AST, aspartate aminotransferase; BUN, urea nitrogen; CK, creatine kinase; Cl, chloride; Cre, creatinine; CRP, C-reactive protein; eGFR, estimated glomerular filtration rate; γGTP, gamma glutamyl transpeptidase; Hb, hemoglobin; Ht, hematocrit; K, potassium; LDH, lactic dehydrogenase; Na, natrium; PLT, platelet; POD, postoperative day; PT, prothrombin time; PT-INR, prothrombin time international ratio; RBC, red blood cell; T-Bil, total bilirubin; TP, total protein; WBC, white blood cell.

## Data Availability

The data that support the findings of this study are available upon request from the corresponding author. The data are not publicly available due to privacy or ethical restrictions.
